# Variability of baroreceptor reflex assessed by tilt table test in a patient undergoing pulmonary vein isolation

**DOI:** 10.1007/s10840-023-01690-x

**Published:** 2023-11-13

**Authors:** Anna Zuk, Roman Piotrowski, Agnieszka Sikorska, Ilona Kowalik, Piotr Kulakowski, Jakub Baran

**Affiliations:** 1 Centre of Postgraduate Medical Education, Department of Cardiology, Grochowski Hospital, Warsaw, Poland; 2 Clinical Research Support Center, National Institute of Cardiology, Warsaw, Poland

**Keywords:** Baroreceptor reflex, Atrial fibrillation, Ablation, Tilt test

## Abstract

**Background:**

The autonomic nervous system (ANS) plays a significant role in atrial fibrillation (AF). Catheter ablation (CA) affects the ANS balance. The assessment of baroreceptor (BR) function is an established method to measure parasympathetic activity; however, it has been rarely used in patients undergoing CA of AF.

**Aims:**

This study is to assess changes in BR function caused by CA and to compare these changes between two different types of CA: point-by-point radiofrequency (RF) versus cryoballoon (CB).

**Methods:**

In this observational, prospective, single center study, 78 patients (25 females, mean age 58 ± 9) with paroxysmal AF and first CA were included: 39 patients (RF group) and 39 (CB group). The BR function was assessed non-invasively using tilt testing and three parameters: event count (BREC) depicting overall BR activity, slope mean depicting BR sensitivity (BRS), and BR effectiveness index (BEI).

**Results:**

The groups did not differ in clinical or demographic data. Before CA, tilting caused a marked decrease in BR function parameters in the whole study group (BREC (29 ± 14.0–50.0 vs 28 ± 9.0–44.0, *p* < 0.068), BRS (10.2 ± 7.1–13.2 vs 5.8 ± 4.9–8.5; *p* < 0.001), and BEI (52.9 ± 39.9–65.5 vs 39.6 ± 23.6–52.1; *p* < 0.001), supine vs tilting, respectively). These changes were similar in the both groups. After CA, BR function decreased in the whole group (BREC 12.0 ± 3.0–22.0 vs 6.0 ± 3.0–18.0, *p* = 0.004; BRS 4.8 ± 3.6–6.8 vs 4.0 ± 3.0–5.8, *p* = 0.014; BEI 18.7 ± 8.3–27.4 vs 12.0 ± 5.1–21.0, *p* = 0.009). BREC was significantly more decreased in the CB vs RF. Similar trend was noted for BRS and BEI.

**Conclusions:**

CA significantly affects BR function. These changes were more pronounced following CB rather than RF CA.

## Background

Atrial fibrillation (AF) affects 2–4% of the adult population with the number of affected individuals expected to grow due to increased life expectancy and concomitant risk factors [1]. Atrial fibrillation is linked to higher morbidity, including heart failure and ischemic stroke [2]. The autonomic nervous system (ANS) is crucial in developing and sustaining AF [3]. Both increased vagal tone and adrenergic drive can trigger AF through the activation of ganglionated plexi (GP) located in the epicardial pad. These GPs contain sympathetic and parasympathetic afferent and efferent neuronal fibers.

Catheter ablation (CA) by means of pulmonary vein isolation (PVI) is an established method for treating AF and can be performed using radiofrequency (RF) current or freezing with cryoballoon (CB). During this procedure, not only PV are isolated but also profound modification of GP, predominantly the parasympathetic part, is achieved. Several studies showed that modification of the ANS improved PVI efficacy and prevented AF recurrences [4, 5]. RF ablation consists of point-by-point applications with a single-tip catheter combined with a three-dimensional (3D) mapping system. This approach allows a significant reduction of fluoroscopy dosage and very accurate placement of RF applications and provides additional important information, such as (left atrium) LA voltage. Ablation with RF relies on thermal energy to induce controlled damage or region-specific necrosis of heart tissue [6]. In turn, CB AF ablation is a “single-shot” approach, which uses hypothermal energy to induce ablation by freezing. Lesion shape with cryoablation is sharper, more homogeneous, and less thrombogenic than lesions resulting with RF [7]. However, the quality of CB AF ablation lesions more depend on left atrial anatomy and is more difficult to choose optimal site for ablation than with RF technique [8]. The effects of PVI on the ANS have been investigated in several studies, usually using heart rate (HR) and heart rate variability (HRV) parameters [9, 10]. However, HRV analysis is a mathematical computation of ANS balance and has several limitations such as dependency on the underlying heart rate and respiratory rate, as well as its interaction with exercise, diet, and medication use [11].

One of the main mechanisms involved in the regulation of the ANS activity is the baroreceptors reflex (BR). It is responsible for controlling arterial pressure through the rapid modulation of autonomic nerves, leading to changes in heart rate. The vagal activity level can be measured by analyzing spontaneous BR reflex. A number of studies showed that baroreflex may have prognostic value in heart disorders like myocardial infarction or heart failure [12, 13]; however, data on BR function in patients undergoing CA of AF are scarce [14–16]. Also, whether the type of energy used during CA (RF vs CB) determines the extent of BR changes is not known.

## Aims

This study is to assess PVI-induced changes in the ANS, measured by BR parameters, in patients undergoing CA using RF or CB.

## Methods

### Study group

This was an observational, prospective, single center study (http://clinicaltrials.gov Identifier: NCT03811639) that enrolled patients admitted for CA of paroxysmal AF between 2016 and 2018. The study was approved by the Local Ethics Committee (No 65/PB/2015), and all participants provided written informed consent.

A total number of 335 patients were admitted to our hospital for AF ablation during this period. The patients were included in the present study if they fulfilled the following inclusion criteria: [1] paroxysmal AF, [2] first CA, [3] sinus rhythm at the time of BRS measurements, [4] no sick sinus syndrome, [5] no implanted pacemaker or defibrillator, [6] no PV anatomy favoring any specific ablation technique (e.g., common trunk of left PV favoring RF), [7] no additional cavo-tricuspid ablation or another atrial linear application, and [8] no evidence of severe heart failure defined as left ventricular ejection fraction < 35%.

### Study outline

Patients were admitted to the hospital one day before the procedure. The blood tests, electrocardiography, and transthoracic echocardiography were performed. Medication, including beta blockers and antiarrhythmic drugs, remained unchanged before and after CA. The transesophageal or intracardiac echocardiography (ICE) was performed to exclude thrombus presence in the left atrial appendage.

The BR function was assessed twice—before CA at 7:00 am on the day of the procedure and 2 days after CA. The BR parameters were measured at rest, while supine, and after tilting (70 degrees, no nitroglycerine challenge). A 25-min tilt test had two phases. In the first phase, patients rested supine for 15 min to stabilize their condition. In the second phase, they were tilted at a 70-degree angle for 10 min. The recordings were performed between the 5th to 10th minutes, while supine and between the 25th second and 5.5th minute while tilting (excluding the first 25 s when the table is tilted). The Task Force Monitor software (CNSystem, 2007, version 2.2, Austria) was used for offline analysis [17].

### Baroreceptor function

**Fig. 1:**
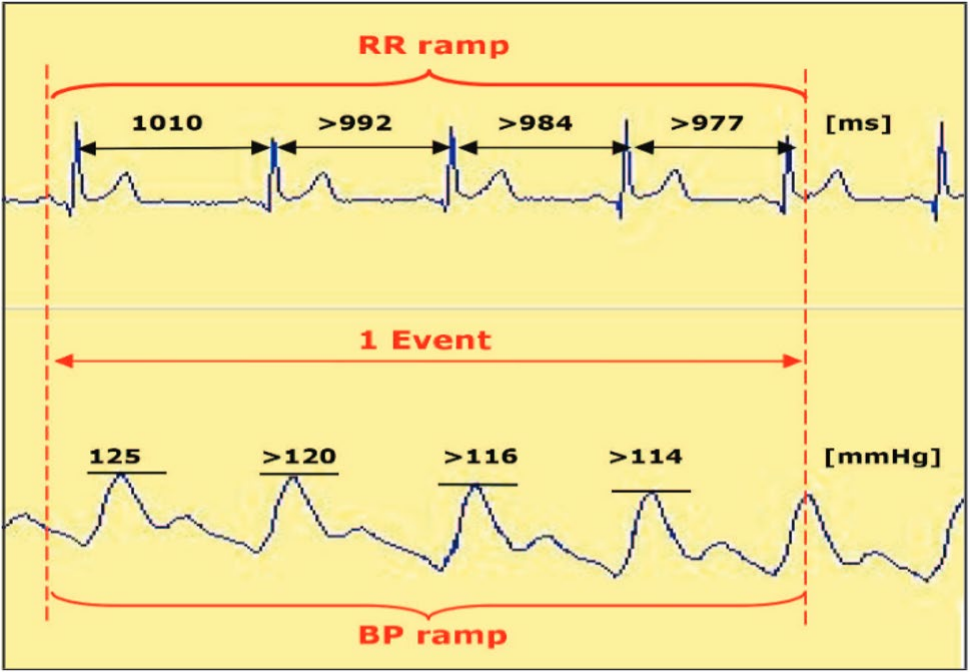
The spontaneous baroreflex sensitivity (BRS) is determined by means of the so-called sequence method [14]

This was calculated by analyzing heart rate (RR interval in ms) and blood pressure (BP) (mmHg) variability. Baroreceptor function was measured by identifying sequences of at least three consecutive heartbeats with either a progressive increase in systolic blood pressure (SBP) and consequent prolongation of the R-R interval or a progressive decrease in SBP and resultant shortening of the R-R interval. The changes in SBP and R-R interval must have been equal to or greater than 1 mmHg over 4 ms. We set the interval between R-R and SBP values at 0 beats and computed the slope of the regression line between the R-R intervals and SBP values for each sequence to determine BRS (in ms/mmHg). An original example of ECG tracings with R-R intervals and SBP values (pletismographic method) is shown in Fig. 1.

Three BR parameters were calculated:

1. Baroreceptor event count (BREC): The overall activity of the baroreceptors was measured by counting the number of events per minute.
2. Baroreceptor slope mean (BRS): The sensitivity of baroreceptors was the extent to which heart rate changes in response to changes in blood pressure and it is measured by the slope of the linear regression between the R-R intervals and the systolic BP values.
3. Baroreceptor effectiveness index (BEI): The effectiveness of baroreceptors was the proportion of events that took place divided by the total number of blood pressure changes.

### Ablation procedures

The RF CA was performed using a single transseptal puncture and Thermocool SmartTouch catheter (Biosense Webster, USA). The CARTO 3 system, ablation index module, and CLOSE protocol were utilized [18]. The ICE was used during all procedures. PVI was confirmed using a diagnostic circular catheter or by pacing from inside the ablation line.

The CB was performed using a flexible 15F sheath (FlexCath Advance, Medtronic, USA) and second-generation CB (Arctic front 2AF281, Medtronic, USA balloon) with a diagnostic Achieve electrode (Medtronic, US) to confirm PVI.

### Statistical analysis

Continuous variables were described as means and standard deviation (if normal distribution data verified by Shapiro-Wilk test) or medians and 25% and 75% percentiles (if skewed distributions). Nominal variables were reported as counts and percentages. Between-group comparisons of the baseline clinical characteristics and diagnostic tests were made using independent sample Student’s *t*-test or Mann-Whitney test, as appropriate, and differences in proportions were compared by means of Chi2 test of independent or Fisher’s exact test, as appropriate. The paired Student’s *t*-test or Wilcoxon signed-rank test was used to compare matched data. The strength of the linear relationships between the changes in baroreceptor reflex values was measured using Pearson’s correlation coefficients (due to the normality of the deltas). The homogeneity of the regression coefficients was checked. One-way analysis of variance with the baseline measurement as the covariance was used to verify the significance of differences between changes in the BRS scores of both study groups (CB and RF). In the case of different slopes of the regression line, interactions were considered. Results were presented as baseline-adjusted mean with 95% confidence intervals. Where a significant interaction is shown, a comparison of the two groups is provided for several baseline values.

All verified hypotheses were two-sided with type I error rate alpha < 0.05. Statistical analyzes and graphs were performed using the SAS v.9.4 statistical package (NC, USA).

## Results

Out of 335 patients, 78 patients (age 58 ± 9 years, 25 females) met inclusion criteria. The RF CA was performed in 39 patients and CB—in the remaining 39 patients. The two groups did not differ in demographic and clinical characteristics (Table 1).

**Table 1:** Comparison of the baseline study population

Parameter	Cryoballoon, *n* = 39	RF ablation, *n* = 39	*P* value
Age (years)	57.5 ± 8.9	58.3 ± 9.9	0.719
Weight (kg)	87.6 ± 13.9	84.3 ± 11.2	0.251
BMI	28.3 ± 3.9	28.2 ± 3.0	0.871
Men	29 (74.4%)	24 (61,5%)	0.225
Heart failure	1 (2.6%)	3 (7.7%)	0.615
Hypertension	20 (51.3%)	28 (71.8%)	0.063
Diabetes	2 (5.1%)	5 (12.8%)	0.431
Stroke	3 (7.7%)	0 (0%)	0.240
Vascular disease	4 (10.3%)	4 (10.3%)	1.00
CHADS2VASc	1 [0; 2]	1 [0; 3]	0.217
Hyperlipidemia	16 (41.0%)	19 (48.7%)	0.495
Kidney disease	0 (0%)	4 (10.3%)	0.115
LA diameter	37.7 ± 4.0	38.3 ± 4.4	0.511
LVEF	60.4 ± 4.7	58.8 ± 6.3	0.198
HASBLED			
0	20 (51.3%)	19 (48.7%)	0.950
1	13 (33.3%)	13 (33.3%)	
2	6 (15.4%)	7 (18.0%)	
Medication			
Beta blockers	26/39 (67%)	28/39 (71%)	0.81
Propafenone	15/39 (38%)	21/39 (54%)	0.26
Sotalol	4/39 (10%)	4/39 (10%)	1.00
Amiodarone	3/39 (8%)	5/39 (13%)	0.71

*BMI* body mass index; *CHADS2-VASc* congestive heart failure, hypertension, age ≥ 75 yo diabetes, stroke, vascular disease, age 65–74, sex category; *HASBLED* hypertension, abnormal liver/renal function, stroke, bleeding history or predisposition, labile INR, elderly, > 65 yo, drugs/alcohol, *LA* left atrium, *LVEF* left ventricle ejection fraction

Mean CB AF ablation time was 150 min with the X-ray time 24.1 min, while mean time RF AF ablation was 250 min with the X-ray time 15.7 min, respectively. The number of lesions to acquire isolation in CB was 6.1 in the time of 1320 s and in RF group 57.6 applications in the mean time of 2835 s. There were two patients with big hematomas about 10 cm in CB group and one patient in RF group. Most of complications that were the small hematomas less than 5 cm in both groups.

### Baroreceptor function before ablation

**Fig. 2:**
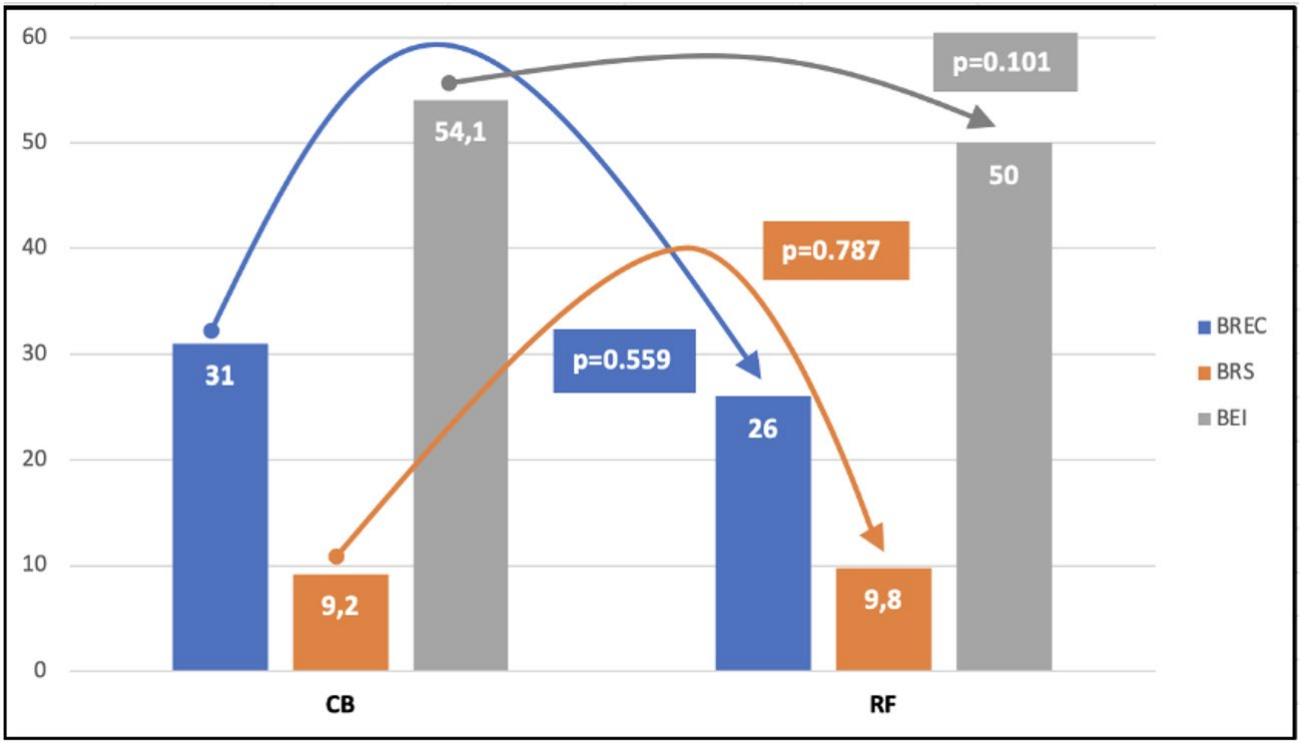
Comparison of baseline baroreceptor parameters before the AF ablation while supine in whole group

**Fig. 3:**
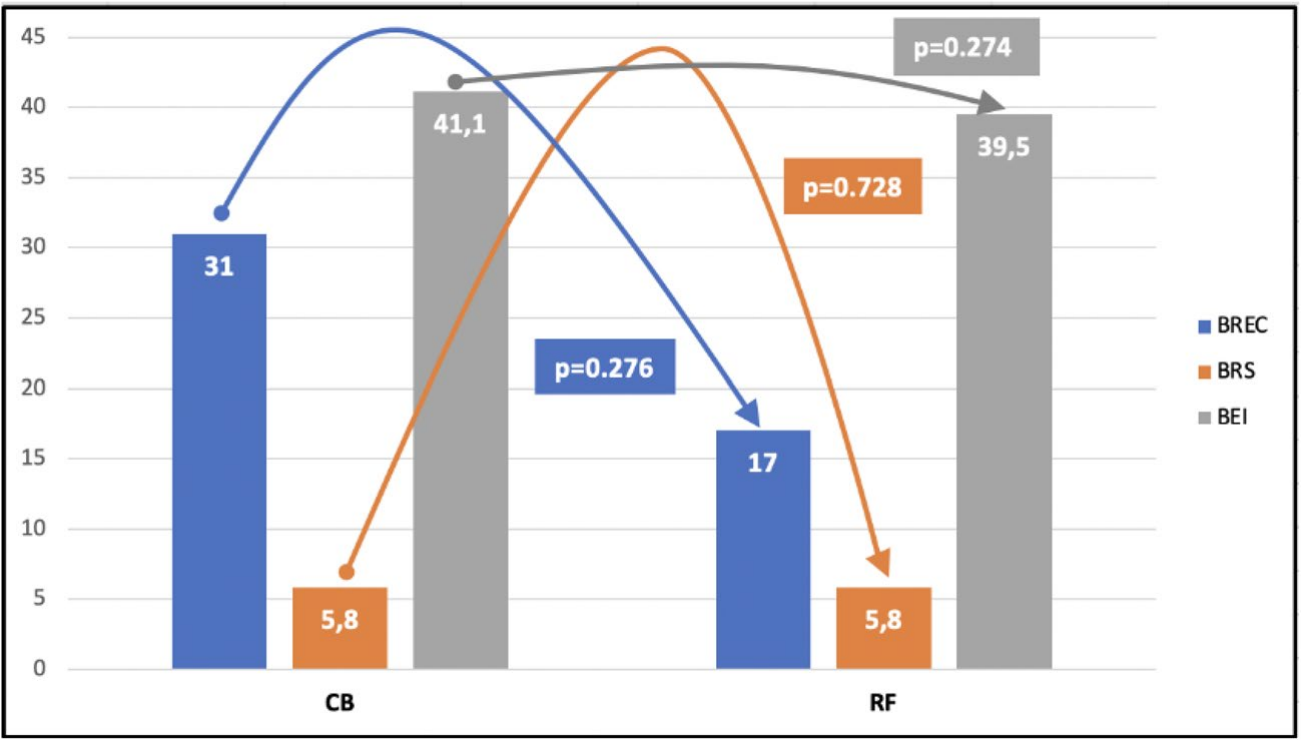
Comparison of baseline baroreceptor parameters before the AF ablation while tilting in whole group

**Fig. 4:**
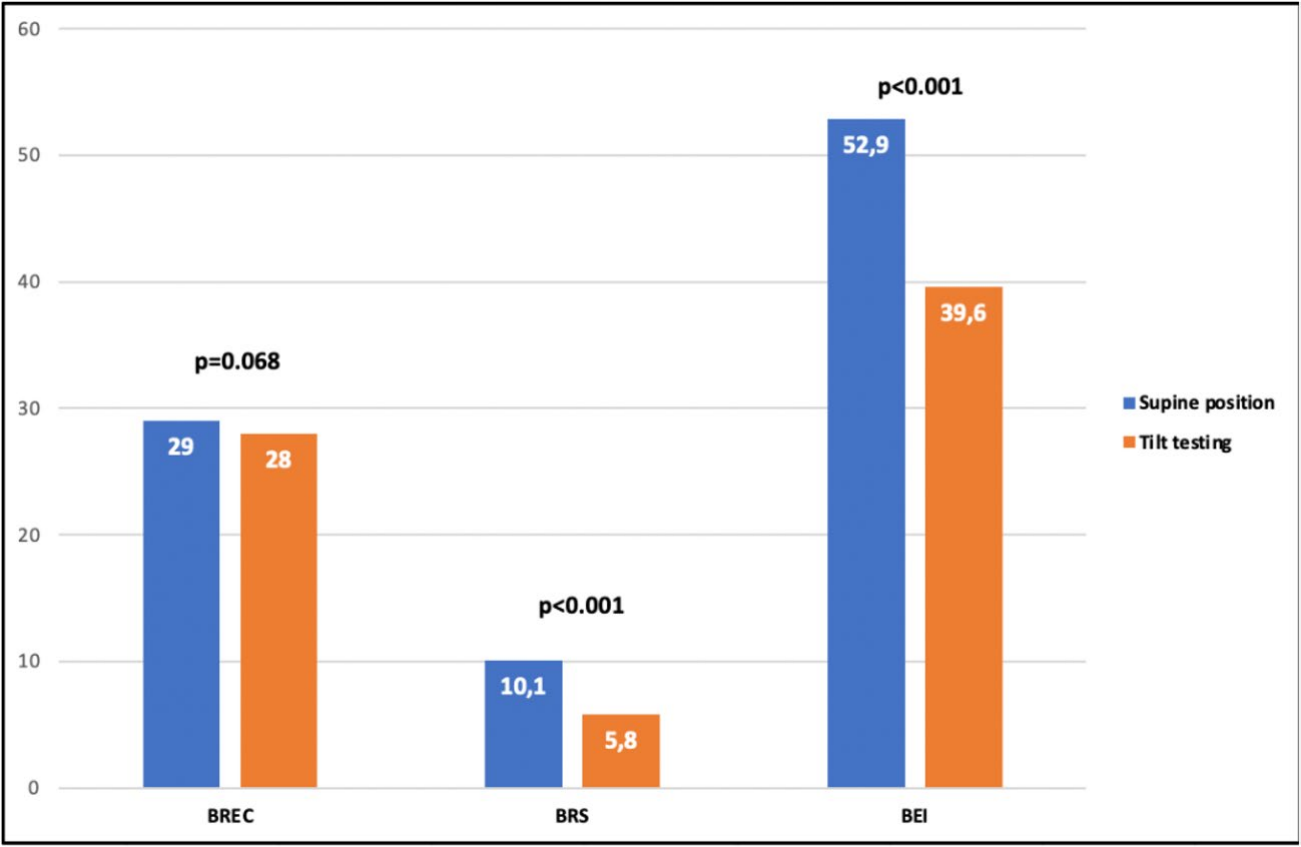
Comparison of baroreceptor parameters while supine versus tilting before the AF ablation in the whole group of patients

The baseline BR parameters, both while supine and after tilting, were similar in both groups (Figs. 2 and 3). Titling resulted in a significant reduction in the BR values, similar in the RF and CB groups (Fig. 4).

### Baroreceptor function after ablation

**Fig. 5:**
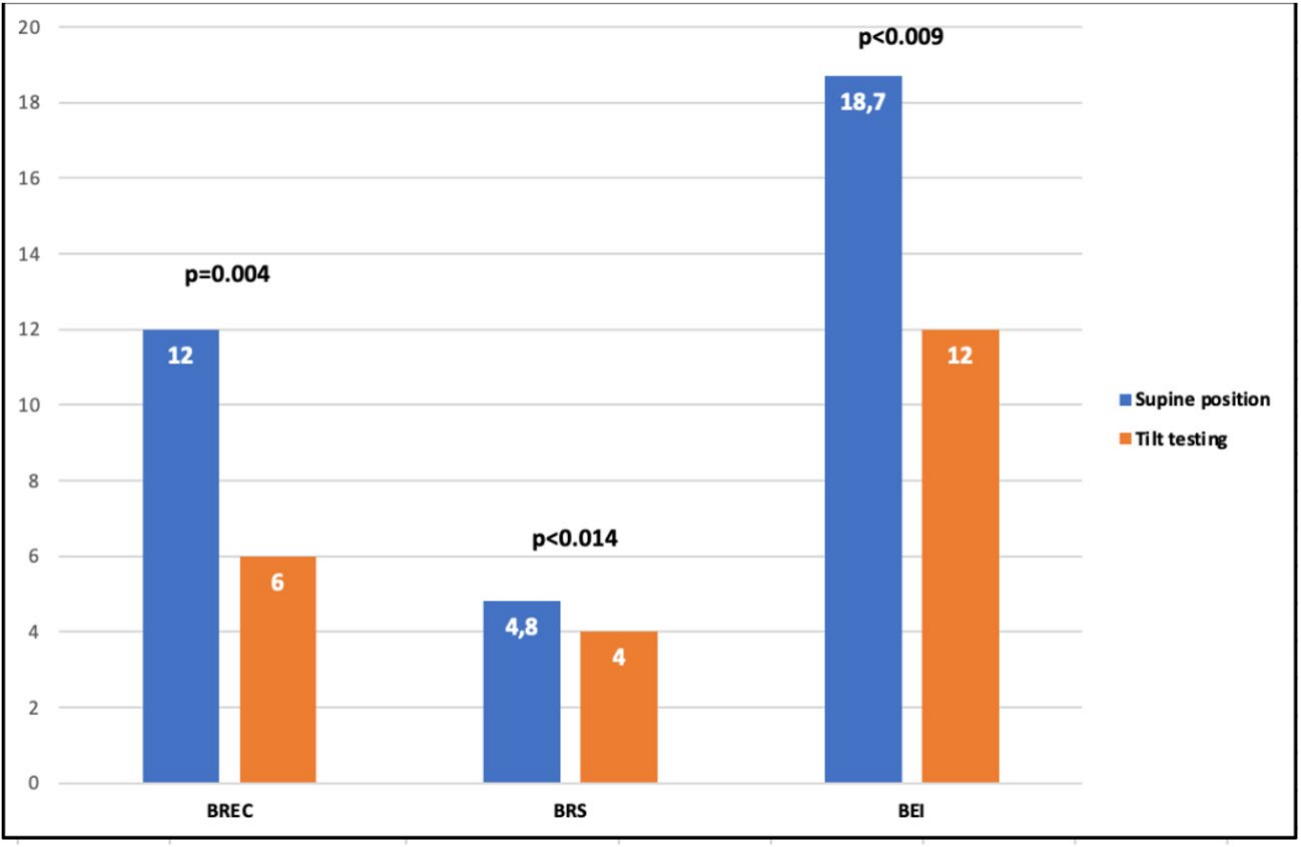
Comparison of baroreceptor parameters while supine versus tilting after ablation in the whole group of patients

After AF ablation, all of the evaluated BR parameters decreased in both groups (Table 2). As before ablation, tilting resulted in a significant reduction of the BRS parameters (Fig. 5). The BR parameters were reduced in both groups while supine and after tilting (Tables 3 and 4). A significant negative correlation was found between the difference in baroreceptor parameters (before and after ablation) and baseline values of baroreceptor parameters in both groups while the supine phase and after tilting. The decrease in BRS parameters was more pronounced in the CB versus RF group (Tables 5 and 6).

**Table 2:** Comparison of BR parameters values before and after the AF ablation in the whole group

	Before the ablation	After the ablation	*P* value
Supine position			
BREC	29.0 [14.0–50.0]	12.0 [3.0–22.0]	< 0.001
BRS	10.2 [7.1–13.2]	4.8 [3.6–6.8]	< 0.001
BEI	52.9 [39.9–65.5]	18.7 [8.3–27.4]	< 0.001
Tilt testing			
BREC	28.0 [9.0–44.0]	6.0 [3,0–18.0]	< 0.001
BRS	5.8 [4.9–8.5]	4.0 [3.0–5.]	< 0.001
BEI	39.6 [23.6–521]	12.0 [5.1–2.1]	< 0.001

*BREC* baroreceptor event count, *BRS* baroreceptor slope mean, *BEI* baroreceptor effectiveness index

**Table 3:** Comparison of BRS parameters before and after CB

	Supine position			Tilt testing		
	Before the ablation	After the ablation	*P* value	Before the ablation	After the ablation	*P* value
BREC	31 [16–46]	15 [9–22]	< 0.001	31 [10–45]	8 [2–20]	0.003
BRS	9.2 [6.5–13.3]	5.2 [3.8–6.8]	< 0.001	5 5.8 [4.8–8.8]	4.1 [3.4–5.0]	< 0.001
BEI	54.1 [41.6–73.2]	20 [11.8–27.6]	< 0.001	41.1 [28.0–56.5]	13.8 [4.2–25.7]	< 0.001

*BREC* baroreceptor event count, *BRS* baroreceptor slope mean, *BEI* baroreceptor effectiveness index

**Table 4:** Comparison of BRS parameters before and after RF

	Supine position		Tilt testing			
	Before the ablation	After the ablation	*P* value	Before the ablation	After the ablation	*P* value
BREC	26 [13–51]	6 [2–19]	< 0.001	17 [6–44]	6 [3–15]	< 0.001
BRS	9.8 [7.1–13.4]	4.1 [3.3–6.7]	< 0.001	5.8 [5.0–8.5]	4.0 [2.8–5.7]	< 0.001
BEI	50.0 [34.2–62.4]	12.5 [5.5–27.0]	< 0.001	39.5 [21.0–50.0]	10.8 [5.5–17.2]	< 0.001

*BREC* baroreceptor event count, *BRS* baroreceptor slope mean, *BEI* baroreceptor effectiveness index

**Table 5:** Table showing the results of correlation coefficients and linear regression coefficients between the difference in BR values (before and after ablation) with baseline BR values in the supine position

Supine position									
	CB				RF				*P*
	Rho	*P* value	Regression coefficient	*P* value	Rho	*P* value	Regression coefficient	*P* value	
BREC	− 0.74	< 0.001	− 0.88 ± 0.11	< 0.001	− 0.61	< 0.001	− 0.45 ± 0.09	< 0.001	0.003
BRS	− 0.80	< 0.001	− 0.89 ± 0.16	< 0.001	− 0.84	< 0.001	− 0.96 ± 0.05	< 0.001	0.615
BEI	− 0.67	< 0.001	− 0.84 ± 0.14	< 0.001	− 0.60	< 0.001	− 0.73 ± 0.15	< 0.001	0.593

*CB* cryoballoon ablation, *RF* radiofrequency ablation, *BREC* baroreceptor event count, *BRS* baroreceptor slope mean, *BEI* baroreceptor effectiveness index

**Table 6:** Table showing the results of correlation coefficients and linear regression coefficients between the difference in BR values (before and after ablation) with baseline BR values in the tilt up position

Tilt testing									
	CB				RF				*P*
	Rho	*P* value	Regression coefficient	*P* value	Rho	*P* value	Regression coefficient	*P* value	
BREC	− 0.80	< 0.001	− 0.997 ± 0.11	< 0.001	− 0.88	< 0.001	− 0.67 ± 0.07	< 0.001	0.015
BRS	− 0.77	< 0.001	− 0.77 ± 0.12	< 0.001	− 0.64	< 0.001	− 0.63 ± 0.13	< 0.001	0.428
BEI	− 0.70	< 0.001	− 0.82 ± 0.11	< 0.001	− 0.62	< 0.001	− 0.76 ± 0.12	< 0.001	0.721

*CB* cryoballoon ablation, *RF* radiofrequency ablation, *BREC* baroreceptor event count, *BRS* baroreceptor slope mean, *BEI* baroreceptor effectiveness index

The CA resulted also in significant differences in both groups in several parameters. The HR increased both in CB and RF group while supine and after tilting after the ablation (Table 7 and 8). The PR interval was statistically decreased after the RF, but it was prolonged in CB group (Table 9).

**Table 7:** Comparison of the HR before and after ablation in CB group

	Supine position			Tilt testing		
	Before the ablation	After the ablation	*P*	Before the ablation	After the ablation	*P*
HR	58 [52–65]	71 [63.5–76.5]	*p* < 0.001	70 [59–76.5]	77 [70.5–85]	*p* < 0.001

**Table 8:** Comparison of the HR before and after ablation in RF group

	Supine position			Tilt testing		
	Before the ablation	After the ablation	*P*	Before the ablation	After the ablation	*P*
HR	60 [56–66.5]	68 [61–78.5]	*p* < 0.001	69 [64–74]	75 [67–81.5]	*p* < 0.001

**Table 9:** Comparison of PR interval before and after CA in CB and RF group

PR interval			
	Before the ablation	After the ablation	*p*
CB	166 [150–190]	168 [142–180.5]	**< **0.001
RF	158 [150–168]	150 [142–166]	0.001

## Discussion

Our study showed that [1] CA AF significantly decreased BR parameters and [2] these effects were more pronounced when using CB rather than RF.

The arterial baroreceptors ensure an input in regulation of BP homeostasis maintaining regulation during changes in body position. The main trigger for the orthostatic reaction is the decompression of baroreceptors as a result of a decrease in BP. Then, there is an increase in BP, mainly diastolic, by increasing peripheral vascular resistance due to vasoconstriction and accelerating HR. Various investigations have been performed to assess the function and activity of baroreceptors [19, 20]. According to the ATRAMI study the reduction in BRS values was clinically significant and was a predictor of cardiac mortality in patients with previous myocardial infarction [13]. The assessment of BRS values was also a predictor of the vasovagal syndrome [21]. BRS has been reported to be a predictor of cardiovascular parasympathetic activity, and its function was depressed in patients with AF [15]. So far, the baroreceptor reflex has not been evaluated as an activity of the autonomic system using tilt testing in a comparison of the effectiveness of two different types of ablation.

Only three studies used BR function to assess ANS changes following CA. All these studies showed reduced BR values after CA; however, they differed from our study. Miyoshi et al. focused on comparison in BR changes between patients with paroxysmal versus persistent AF and showed that baseline BR activity was more depressed in patients with persistent AF [15]. Catheter ablation depressed BR function irrespective of the type of AF, with a greater effect in patients with paroxysmal than persistent AF. They also suggested that depressed BR, also known as intrinsic parasympathetic nervous dysfunction, may contribute to the higher recurrence rate after CA in patients with persistent AF [15]. Kondo et al. showed that a lack of decrease in BR after the RFCA may predict procedural failure [14]. Also, Styczkiewicz et al. showed, using Valsalva maneuver as a trigger for the BR reflex, that successful PVI may lead to transient autonomic alterations reflected by a reduction in pulse interval variability and BR reflex, with more prolonged changes in the Valsalva ratio [16].

Inhibition of the BR reflex following CA reflects denervation of the heart, mainly the parasympathetic part, which may have clinical implications. Denervation of the GPs during PVI leads to a reduction of AF recurrences [22]. Multiple studies comparing PVI and PVI + GP ablation showed that the effectiveness of PVI + GP ranged between 50 and 91% [23, 24]. Stojadinovic et al. showed that AF ablation alone around PVs caused complete parasympathetic denervation [25]. The mechanism by which BRS is lowered in AF patients is not clear. It is well known that arrhythmia leads to enlargement of LA due to fibrosis, remodeling, and inflammatory processes, which in turn can affect the function of GPs located in the LA. Patients with AF are also known to have enhanced endothelial dysfunction, leading to the impaired vascular function [26, 27].

AF ablation, be it RF or CB, alters autonomic control of the heart and predominantly decreases the parasympathetic drive to the heart by ablation ganglionated plexi localized in the atrial epicardial fat [28]. In fact, apart from PVI, vagal denervation is probably one of most important mechanisms associated with successful outcome, especially in patients with so-called vagally mediated AF. The long-term effects of ablation-induced vagal inhibition and impairment of BRS, as we have shown in the present study, are not known and may rise some concern in view of protective effects of parasympathetic part of the autonomic nervous system on the heart. However, AF ablation has been performed worldwide for more than 20 years, and there is not a single report suggesting increased mortality or susceptibility to dangerous ventricular arrhythmias following this procedure. Longer follow-up duration is needed to address this issue.

Based on correlation coefficients in our study, CB had a greater impact on ANS denervation than RF, probably due to deeper penetration of freezing energy in the myocardial tissue. It has been shown that cryoenergy may not only affect atrial tissue but also penetrate to neighboring structures, causing collateral damage [29].

In our study, we used tilt testing to trigger the BR reflex. Tilt testing is an established method for evaluating BR. The main idea behind the test is that due to the erect position, the baroreceptor reflex leads to vasodilatation or vasoconstriction which in turn causes HR changes that reflects cardiac autonomic reflex [30–32].

### Strengths of the study

The strengths of the study are as follows:

1. Well-defined and homogenous population of patients with paroxysmal AF.
2. Use of BR to evaluate CA-induced BR changes.
3. Use of tilt testing as a trigger for BR assessment.
4. Confirmation of BR attenuation following CA of AF.
5. Demonstrating that CB leads to a greater decrease in BR than RF.

### Limitations

Firstly, the present study included a relatively low number of patients, however, sufficient to demonstrate CA-induced changes in baroreflex and differences between RF and CB. Secondly, the number of patients was too low to compare the predictive value of BR reflex changes on the outcome. Thirdly, it was an unblinded and non-randomized study. Fourthly, due to lack of data regarding long-term BRS function, it is not possible to predict the BRS function with the clinical outcome of ablation. And finally, the fact that baroreflex function was only assessed shortly after ablation and not at a later time point, thus not permitting evaluation of longitudinal changes.

## Conclusions

The ablation of the AF substrate significantly affected the functions of ANS based on the changes in the baroreceptor system. Both after CB and RF the function of the baroreceptor reflex was reduced in all analyzed parameters, both in the supine phase and after tilt provocation. The larger changes were recorded after CB which may be related to a deeper interference in the atrial tissues, and thus in the ganglia located there.
